# What have we learnt from histology about the efficacy of coronary imaging modalities in assessing plaque composition?

**DOI:** 10.3389/fcvm.2025.1507892

**Published:** 2025-01-24

**Authors:** Nathan Angelo Lecaros Yap, Zahid Khan, Xingwei He, Jae-Geun Lee, Soe Maung, Kimberley R. Morgan, Tingquan Zhou, Helle Precht, Patrick W. Serruys, Hector M. Garcia-Garcia, Yoshinobu Onuma, Sean Hynes, Sebastian Kelle, Anthony Mathur, Andreas Baumbach, Christos V. Bourantas

**Affiliations:** ^1^ Device and Innovation Centre, William Harvey Research Institute Queen Mary University, London, United Kingdom; ^2^ Barts Heart Centre, Barts Health NHS Trust, London, United Kingdom; ^3^ Medical Education, University of South Wales, Wales and University of Buckingham, Buckingham, United Kingdom; ^4^ Conrad Research Center, Radiography Education, University College Lillebælt, Odense, Denmark; ^5^ Faculty of Medicine, National Heart & Lung Institute, Imperial College London, United Kingdom; ^6^ Department of Cardiology, National University of Ireland, Galway, Ireland; ^7^ Interventional Cardiology Department, MedStar Washington Hospital Center, Washington, DC, United States; ^8^ Department of Pathology, National University of Ireland, Galway, Ireland; ^9^ Department of Cardiology, Radiology and Intensive Care Medicine, Deutsches Herzzentrum Der Charite, Berlin, Germany

**Keywords:** intravascular ultrasound, optical coherence tomography, near-infrared spectroscopy, histology, hybrid intravascular imaging

## Abstract

Accurate evaluation of coronary artery pathology is essential for risk stratification and tailoring appropriate treatment. Intravascular imaging was introduced for this purpose 40 years ago enabling for the first time *in vivo* plaque characterization. Since then, several studies have evaluated the efficacy of the existing intravascular imaging modalities in assessing plaque pathology and composition and their potential in guiding intervention and predicting vulnerable plaques. Today it is known that intravascular imaging is an indispensable tool in percutaneous coronary intervention planning, but the existing modalities have a limited efficacy in predicting lesion vulnerability; a fact that should be attributed to their advantages and limitations in accurately assessing morpho-pathological features that are common in advanced atherosclerotic plaques. This review aims to provide a comprehensive evaluation of the performance of intravascular imaging in characterizing plaque phenotypes using histology as a reference standard; it summarizes the studies comparing the available invasive imaging techniques against histology, discusses the findings and limitations of these studies and highlights the potential of novel intravascular imaging approaches that were introduced for a more complete and comprehensive evaluation of plaque pathobiology.

## Introduction

Andreas Grüntzig not only introduced percutaneous coronary intervention (PCI) establishing a less invasive alternative for treating coronary artery disease but also showed the feasibility of the *in vivo* instrumentation of the coronary arteries with pressure catheters to measure vessel physiology (1). His pioneering work inspired engineers towards the development of intravascular catheters that allow real-time evaluation of plaque pathology and physiology enabling a better understanding of atherosclerotic evolution. Yock, taking advantage of his previous research experience in the navy, was the first that built an invasive coronary imaging modality—i.e., the intravascular ultrasound (IVUS)—that provided *in vivo* assessment of plaque characteristics (2). The preliminary applications of IVUS in clinical practice generated hopes that invasive imaging would be able to detect vulnerable plaques and guide therapy in patients with obstructive coronary artery disease and drive research and industry towards the development of alternative invasive imaging modalities that could provide additional information about plaque morphology. Optical coherence tomography (OCT) was introduced a decade after the first applications of IVUS and near-infrared spectroscopy (NIRS) combined with IVUS a decade later to allow for the first time *in vivo* detection of plaque burden and biochemical composition.

These three modalities have been extensively used in the clinical practice and research to evaluate atheroma characteristics and optimise treatment planning and today there is robust evidence that support its routine use in complex PCI to reduce major adverse cardiac events (MACE) (3–5). Conversely, studies that have explored the value of IVUS, OCT and NIRS-IVUS to detect vulnerable lesions have demonstrated that these modalities can detect plaques that are prone to progress and cause events, however, with a limited accuracy and a positive predictive value that is <20% casting doubts about their value in risk stratification and vulnerable plaque detection (6). The limited efficacy of invasive imaging to predict atherosclerotic disease progression has been attributed to the complex pathophysiology of the disease that is regulated by systemic factors, the coagulation properties of the blood, local factors such as the distribution of the haemodynamic forces, and also to the fact that invasive imaging has limitations in accurately detecting specific morphological features that are seen in vulnerable plaques (7). The latter factor has driven research towards the design of advanced multimodality imaging catheters for a more comprehensive and complete evaluation of atheroma phenotypes.

This review paper aims to provide an overview of the histology studies that examined the performance of IVUS, OCT, and NIRS-IVUS in characterising plaque pathology, present the novel invasive imaging technologies introduced to overcome the limitations of the first intravascular imaging catheters, and summarize the results of preliminary histology studies that tested the performance of these approaches in detecting plaque features associated with increased vulnerability.

## Intravascular ultrasound imaging

### Grayscale intravascular ultrasound

**Figure 1:**
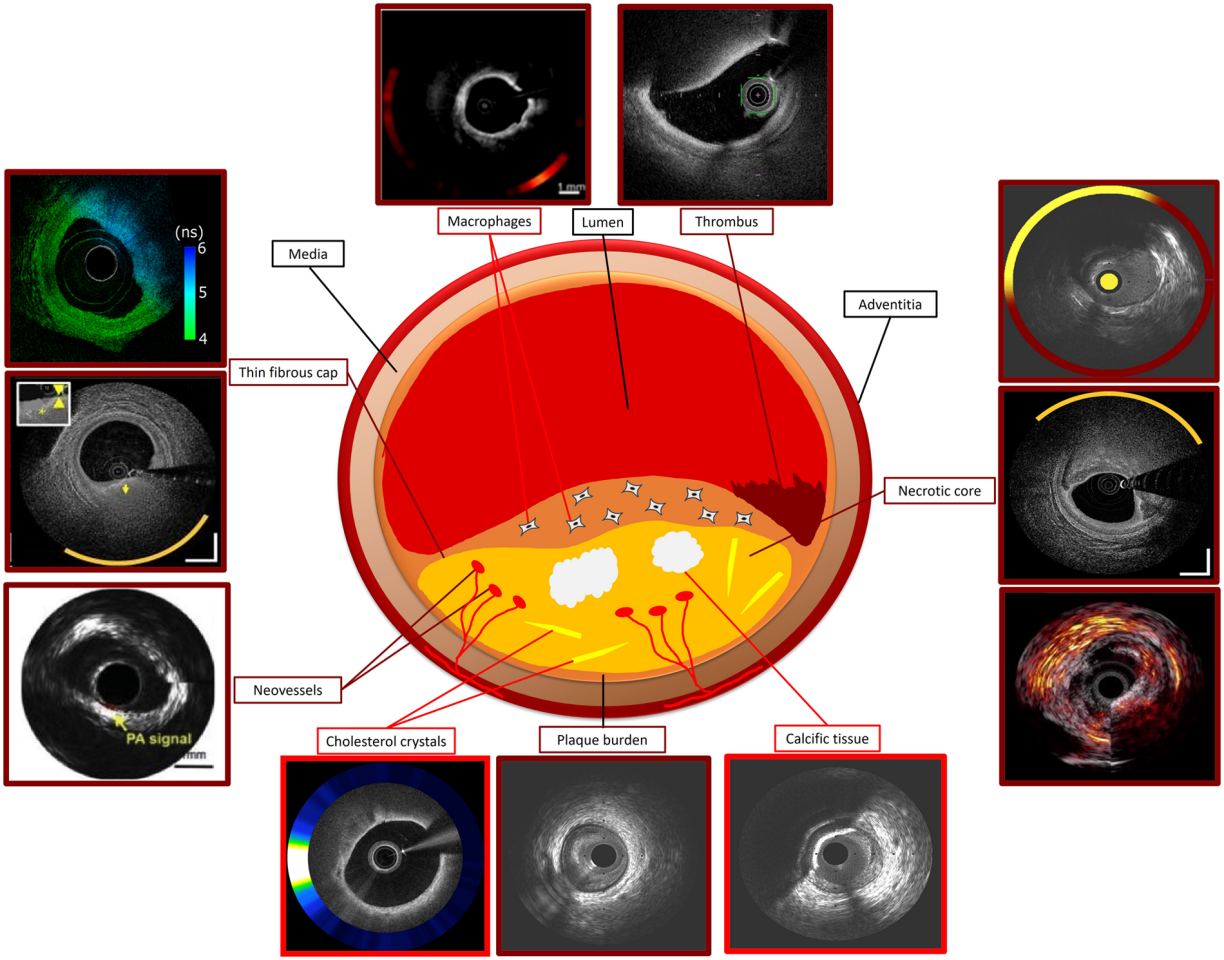
Ideal standalone and hybrid intravascular imaging modality for assessing plaque characteristics using histology as reference standard. Modalities with good performance in detecting a specific plaque feature are indicated with dark red and those with moderate performance with a bright red colour. Photoacoustic image of necrotic core courtesy of Jansen et al. (10.1016/j.pacs.2013.11.003). OCT-NIRS image of thin fibrous cap courtesy of Ali et al. (10.1016/j.jscai.2024.101344). OCT-NIRF image of macrophages and cholesterol crystals, FLIm-OCT image of thin fibrous cap, IVPA-IVUS image of neovessels, NIRS-IVUS OCT-NIRS and IVPA-IVUS images of necrotic core courtesy of Tufaro et al. (10.1016/j.jcin.2024.07.007).

**Figure 2:**
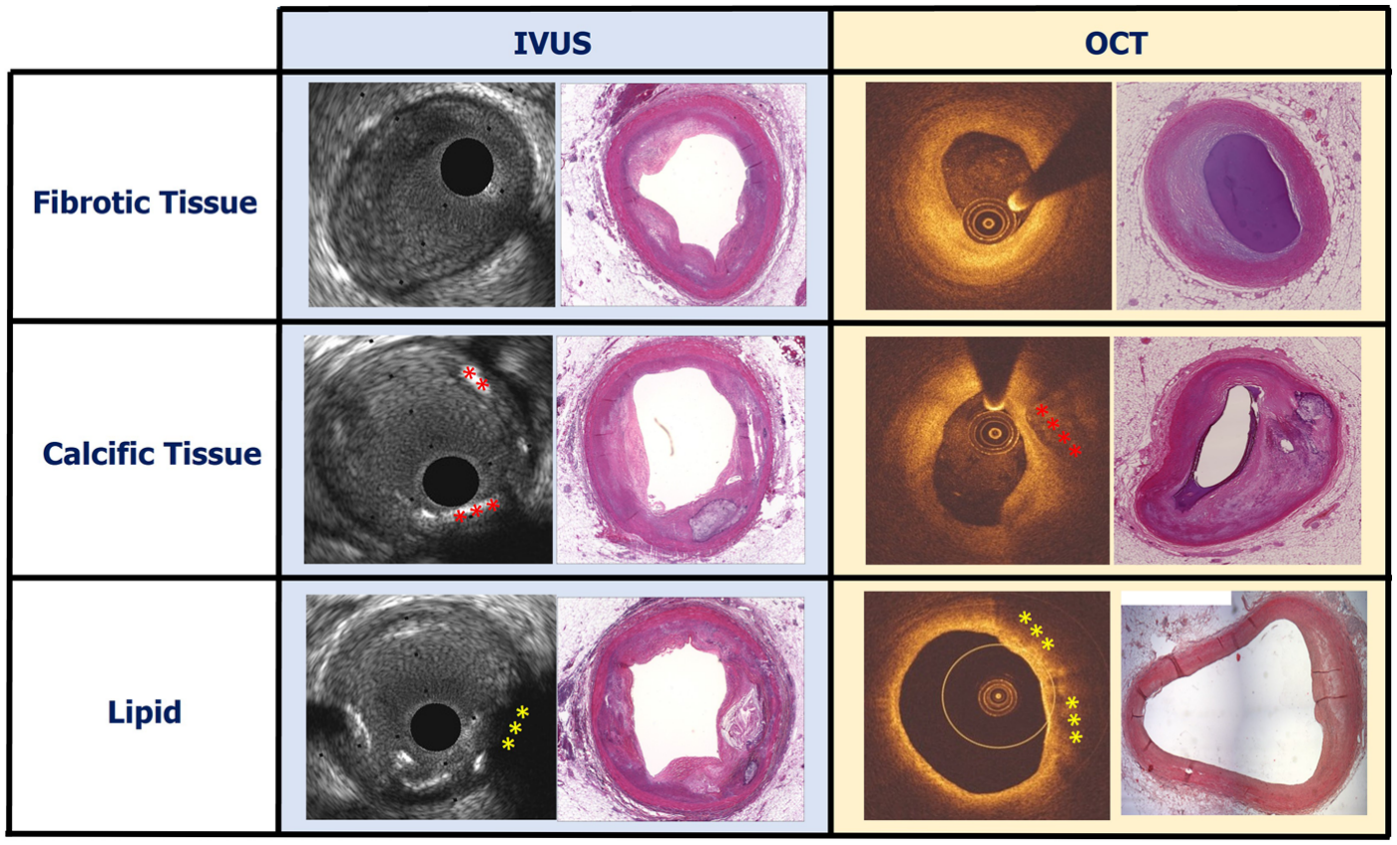
Case examples showing a fibrotic, calcific and lipid-rich plaque on IVUS and OCT images and the corresponding histological sections stained with haematoxylin and eosin. Calcific plaque on IVUS and OCT is indicated by red asterisks and lipid on IVUS and OCT is indicated by yellow asterisks.

The ability of IVUS to assess atherosclerotic disease severity and quantify the lumen and plaque dimensions became apparent from the first validation studies comparing the estimations of this modality against histology (8). However, the performance of IVUS to accurately characterise plaque composition and detect the presence of lipid tissue has been questioned. Preliminary reports have shown that the intensity of the ultrasound signal within the plaque provides information about tissue composition with areas of grayscale intensity lower than that of the adventitia corresponding to lipid tissue, those with areas similar to that of the adventitia to fibrotic, and those with higher than that of the adventitia and acoustic shadowing behind corresponding to calcific tissue (Figures 1, 2) (9). This tissue characterization approach has been incorporated into a user-friendly software called echogenicity, that allows automated characterization of plaque composition based on the grayscale intensity of the pixels constituting the plaque. A histology study in pigs has shown that this approach enables accurate characterization of the plaque composition however the first validation in cadaveric human hearts cast doubts about its efficacy in detecting plaque components (10, 11).

More recent studies, however, have provided evidence supporting the validity of pixel intensity in characterizing plaque types. A report including 2,294 IVUS frames matched with histology showed that the presence of echo-lucent plaques (defined as plaques with a pixel intensity smaller than that of the adventitia) and of attenuated plaque (defined as plaques with signal attenuation without the presence of calcific tissue) was associated with the presence of a necrotic core on histology. However, these features had limited efficacy in detecting a necrotic core with a low sensitivity (56.2% for the attenuated plaques and 20.5% for the echolucent plaques) but a high specificity (94.7% for the attenuated plaques and 90.4% for the echolucent plaques) (Table 1) (12).

**Table 1:** Histology studies assessing the efficacy of intravascular imaging modalities in characterizing plaque morphology using human histology data as reference standard.

Study	Objectives	Sample size	Results
Greyscale IVUS			
Gussenhoven et al. (8)	To examine the ability of 40 MHz IVUS imaging to assess plaque composition	11 human arterial segments form carotid and iliac arteries were studied and diseased section was analysed	• Hypoechoic areas, soft echoes and bright echoes areas corresponded to lipid, fibromuscular and dense fibrous tissue/calcific deposits respectively on histology • A good correlation was noted between the thickness of the lesion measured from the histology and IVUS images
Palmer et al. (84)	To examine the reproducibility and the agreement between IVUS and histology for plaque composition	50 cross-sections selected from 30 human coronary arteries were analysed	• IVUS had a high intra-observer and inter-observer reproducibility in assessing plaque types (*κ*: 0.89, κ: 0.87) • IVUS correctly detected 89% of the plaque types identified by histology • IVUS had an excellent specificity for detecting fibrotic, lipid and calcific plaques (95%, 96% and 92% respectively) but the sensitivity varied for these three plaque types (78%, 94% and 100% respectively)
Pu et al. (12)	To examine the association between specific morphological characteristics on IVUS (echo attenuation, and intraplaque echo-lucent zones) and plaque composition in histology	2,294 artery segments, 2 mm in size, from 151 human coronary arteries underwent IVUS, NIRS imaging and histological analysis	• IVUS detected echolucent plaques in 10.5% of the cases and echo-attenuated plaques in 18.3%. • Most echo-attenuated plaques (91.4%) corresponded to either a FA or PIT with a lipid pool. Almost all segments with superficial echo-attenuated plaques indicated FA with advanced necrotic core. • Echo-lucent plaques corresponded in 78.6% of the cases to necrotic cores or lipid pools but had a smaller necrotic core or lipid pool compared to echo-attenuated plaques (thickness 0.51 vs. 0.70, *P* < 0.001 and arc 74.5°vs. 90°, *P* < 0.001) • The sensitivity and specificity of echo-attenuated and echolucent plaques to detect large lipid cores were 56.2%, 20.5% and 94.7% and 90.4% respectively
OCT			
Plaque composition			
Yabushita et al. (26)	To establish objective image criteria for characterizing plaque composition in OCT using histology as a reference standard	357 matched OCT and histology cross-sections from the coronary, carotid arteries and the aortas were included in the study; 50 were used for training and 307 for testing	• Fibrous plaques in histology had in OCT a homogeneous, highly backscattering signal, the fibrocalcific plaques appeared as signal-poor regions with sharply delineated borders and the lipid-rich plaques as diffusely bordered, signal-poor regions • The sensitivity and the specificity of the two experts who analysed the OCT data in detecting plaque types were 79% and 71% for the 1st and 97% and 98% for the 2nd for the fibrotic 95%, 96% and 97% and 97% for the fibrocalcific and 90% and 94% and 90% and 92% the lipid-rich plaques respectively
Kawasaki et al. (20)	To validate the diagnostic accuracy of grayscale IVUS, IB-IVUS, and OCT in characterising plaque types	128 diseased segments obtained from 42 coronary arteries from 17 human cadavers were analysed	• OCT had a higher sensitivity and specificity compared to IB-IVUS and IVUS in assessing fibrotic (sensitivity: 98% vs. 94% vs. 93%, specificity: 94% vs. 84% vs. 61%) and lipid (sensitivity: 95% vs. 84% vs. 67% and specificity 98% vs. 97% vs. 95%) but there was no difference for the calcific tissue (sensitivity: 100% vs. 100% vs. 100% and specificity 100% vs. 99% vs. 99% respectively)
Rieber et al. (28)	To compare the diagnostic accuracy of OCT and IVUS in characterising plaque composition using histology are the reference standard	Histological sections from 17 coronary arteries assessed by IVUS and OCT were taken and divided into 4 quadrants (323 quadrants were included in the analysis)	• The sensitivity and specificity of the two modalities for identifying a normal vessel wall was 91% and 88% for OCT vs. 55% and 79% for IVUS, to detect fibrotic plaques 64% and 88% vs. 63% and 59% and to identify lipid plaques 77% and 94% vs. 10% and 96% while for calcified plaques it was 67% and 97% vs. 76% and 98% respectively
Kume et al. (29)	To compare the efficacy of OCT and IVUS in assessing plaque types using histology as a gold standard	166 sections from 108 coronary arterial segments obtained from 40 human cadaveric hearts were included in the study	• OCT and IVUS were equally effective in detecting fibrotic (sensitivity 79% vs. 88%, specificity 99 vs. 86% respectively) and calcific plaques (sensitivity 96% vs. 98%, specificity 88% vs. 96% respectively) whereas OCT enabled more accurate detection of the lipid tissue (sensitivity 85% vs. 59% and specificity 94% vs. 97% respectively)
Manfrini et al. (92)	To assess the efficacy of OCT in identifying plaque morphology	68 histological cross-sections assessed by OCT were included in the study	• OCT had 45% sensitivity and 83% specificity for detecting atheroma and FA, 83% sensitivity and 82% specificity for detecting fibrotic lesions, 68% sensitivity and 76% specificity for detecting calcific and 100% sensitivity and specificity for identifying complex lesions • Sources of error in plaque characterization included the poor penetration depth of OCT that did not allow detection of the lipid and calcium behind thick fibrous caps and the inability of the modality to differentiate lipid from calcium
Gruslova et al. (46)	To assess the accuracy of core labs in identifying plaque components and phenotypes in OCT images using histology as a reference standard	51 OCT sequences obtained from 43 human hearts were provided to 7 core labs and their estimations were compared with reference to ex-vivo histology	• The core labs were able to identify calcific, fibrotic and lipid tissue on with a good/moderate agreement (kappa of 0.83, 0.93, and 0.58 respectively) • The core labs had a weak performance in detecting vulnerable plaque features and in particular TCFA, necrotic core, macrophages, lipid pools, and calcific nodules with kappa values of 0.22, 0.22, 0.39, 0.35, and 0.50 respectively
Plaque micro-characteristics and thrombus			
Tearney et al. (39)	To investigate the efficacy of OCT in identifying macrophages in fibrous caps using the standard deviation of the OCT signal intensity	OCT images portraying 26 lipid-rich atherosclerotic plaques (19 located in the aorta and 7 in carotid bulbs) from 17 cadavers were analysed	• A high correlation was noted between the OCT signal and histology for the macrophage density (*r* = 0.84, *P* < 0.0001). • A negative correlation was noted between OCT and histological measurements of smooth muscle actin density (*r* = −0.56, *P* < 0.005)
Phipps et al. (41)	To test the performance of OCT in detecting macrophages in the entire plaque	1,559 OCT frames obtained from 14 human coronary arteries from 10 hearts were co-registered with histological cross-sections	• A dedicated algorithm was used to define bright spots in OCT and these estimations were compared with histology • Macrophages on histology were present in 57% of bright-spot-positive regions in OCT. The sensitivity and specificity of the algorithm to detect bright spots was 80% and 49% respectively • Additional causes of bright spots on OCT were cholesterol crystals, layered plaques, the intima-media or media-adventitia border, the calcific-lipid, the fibrous-lipid or the calcific-fibrous border and the neo vessels-media border
Di Vito et al. (40)	To examine the capability of OCT in identifying macrophages in the entire plaque	43 histological and OCT sections from 18 atherosclerotic plaques and 25 matched sections from non-diseased segment were included	• A two-step algorithm that incorporated the OCT signal variance information and a granulometry index was proposed for detecting the inflamed regions in the plaque • The sensitivity and specificity of this algorithm for detecting macrophages was 100% and 96.8% respectively
Katayama et al. (43)	To examine the performance of OCT in detecting cholesterol crystals using histology as reference standard	Coronary segments (*n* = 45) with length 10–20 mm were assessed by OCT and its estimations were compared to histology	• The sensitivity and specificity of OCT for detecting cholesterol crystals was 68% and 92% respectively
Jinnouchi et al. (44)	To assess the efficacy of OCT in detecting cholesterol crystals	559 OCT frames from 45 autopsy cases were co-registered with histology; out of these 117 sections showed a necrotic core with cholesterol clefts	• OCT had a weak sensitivity but a high specificity for detecting cholesterol crystals (25.6% and 100.0%, respectively) • The presence of fibrous plaques and >3 layers of cholesterol crystals arranged one on top of another were associated with increased efficacy of OCT in detecting cholesterol crystals
Kume et al. (45)	To assess the feasibility of time-domain OCT for detecting neo-vessels	55 coronary plaques from 31 human cadavers were imaged by time domain OCT and the OCT frames were matched with histology	• OCT detected neo-vessels in 11 out of the 21 plaques that had neo-vessels in histology. • The sensitivity and specificity of time-domain OCT to detect neo-vessels was 52 and 68% respectively
Shimokado et al. (93)	To assess the value of OCT in identifying healed coronary plaques	144 OCT sections portaying diseased vessels (≥50% stenosis) were co-registered with histology; 30 were employed to define morphological features of healed plaques in histology and the remaining for testing	• A healed plaque had in OCT heterogeneous signal-rich layers with a different signal density • The sensitivity, specificity, positive predictive value, and negative predictive value of OCT to detect healed plaques was 81%, 98%, 93%, and 93%, respectively
Kume et al. (94)	To test the efficacy of OCT in detecting coronary thrombi	108 segments with length 5 cm from the proximal epicardial coronary arteries obtained from 40 human cadavers were assessed by OCT and the obtained images were matched with histology	• White and red thrombi were noted in 18% and 17% of the 108 arterial segments, respectively • OCT identified white thrombi as low-backscattering protrusions and red thrombi as high-backscattering protrusions with a signal-free shadowing behind • OCT had a high efficacy in classifying thrombus type (sensitivity 90%, and specificity 88%)
Eriksen et al. (95)	To assess the value of OCT in characterizing thrombus type classified according to erythrocyte content and age	42 aspirates containing thrombus from 66 STEMI patients were analysed	• Red thrombi were seen in 11 cases, white in 21 and the remaining 10 were classified as mixed thrombi; 36 aspirates had fresh, 7 lytic and 8 organized thrombi • OCT was unable to predict erythrocyte or platelet content and thrombus age
Combined IVUS-OCT imaging			
Goderie et al. (96)	To assess the efficacy of VH-IVUS, OCT and combined VH-IVUS and OCT imaging in detecting plaque types	36 matched IVUS, OCT and histological sections from 9 vessels were included in the analysis	• OCT correctly detected plaque types in 66.6% of the cases, VH-IVUS in 69.4% and combined VH-IVUS and OCT in 75% of the cases
Fujii et al. (30)	To assess the efficacy of OCT, IVUS and the combined IVUS and OCT imaging in identifying TCFA	685 plaques obtained from 165 coronary arteries from 65 human hearts were assessed by IVUS and OCT and the acquired images were matched with histology	• The sensitivity, specificity, positive, negative predictive value, and overall diagnostic accuracy of IVUS in detecting TCFA was 92%, 93%, 19%, 99%, and 93%, respectively • OCT had a higher positive predictive value (41%) but similar sensitivity specificity, negative predictive value and accuracy with IVUS (100%, 97%, 100%, and 98%, respectively) • Combined OCT & IVUS imaging significantly improved the positive predictive value of standalone OCT and IVUS in detecting TCFA (69%) while the sensitivity, specificity, negative predictive value and accuracy remained high (92%, 99%, 99%, and 99%, respectively)
Brown et al. (97)	To assess the efficacy of IVUS-VH, OCT imaging and their combination in detecting TCFA	258 histological cross-sections from the coronary arteries of 14 hearts that were assessed by VH-IVUS and OCT were included in the study	• The sensitivity and specificity of VH-IVUS and OCT in detecting TCFA was moderate 63.6% and 78.1% for VH-IVUS and 72.7% and 79.8% for OCT • The diagnostic performance of combined VH-IVUS and OCT imaging was higher than standalone imaging (sensitivity 68.2% and specificity 91.5%)
Nakano et al. (21)	To assess the performance of OCT in recognizing plaque morphologies and examine the additive values of IB-IVUS in detecting vulnerable plaque	360 cross-sectional images obtained from 14 autopsy hearts (27 coronary arteries) were assessed by OCT and IB-IVUS and co-registered to histology	• OCT could detect 14 of the 18 TCFA, with a moderate positive predictive value (60.9%, κ: 0.664 AUC: 0.88) • When IB-IVUS was combined with OCT the positive predictive value of intravascular imaging dor detecting TCFAs was improved to 100.0% (κ = 0.704; AUC: 0.93)

AUC, Area under curve; FA, Fibroatheroma; IB-IVUS, Integrated backscatter IVUS; IVUS, Intravascular ultrasound; OCT, Optical coherence tomography; NIRS, Near Infra-Red Spectroscopy; PIT, Pathological intimal thickening; STEMI, ST-elevated myocardial infarction; TCFA, Thin-capped fibroatheroma; VH-IVUS, Virtual histology intravascular ultrasound.

To overcome the limitations of greyscale IVUS to assess plaque composition, processing of the amplitude and the frequency of the reflected IVUS has been proposed. Three different approaches have been introduced for the radiofrequency analysis of the IVUS data: virtual histology (VH)-IVUS that uses autoregressive models to classify tissue types, integrated backscatter (IB) analysis that applies fast Fourier transformation to process the IVUS data, and iMAP that also relies on the identification of 40 spectral features in IVUS using autoregressive modelling and their comparison against the IVUS data obtained from typical plaques (13).

### Virtual histology intravascular ultrasound

VH-IVUS can differentiate four tissue types: necrotic core, fibrofatty, fibrous and calcific tissue that are colour-coded displayed (red, light green, green and red and white). The first studies assessing the efficacy of VH-IVUS to characterise plaque composition showed promising results (14, 15). The tissue distribution in VH-IVUS was used to define for the first time *in vivo* plaque phenotypes whose vulnerability was tested in subsequent prospective studies (16).

However, more recent histology studies in porcine models published over the last decade cast doubts about the efficacy of VH-IVUS in assessing tissue composition (10, 17). This was also confirmed in a study performed in hearts obtained from patients listed for transplantation (*n* = 642 matched cross sections) which showed that VH-IVUS has a high sensitivity (94%), but low specificity (53%) and positive predictive value (48%) in detecting necrotic core. Today this technology has been withdrawn and is not clinically available (18).

### Integrated backscatter intravascular ultrasound analysis

Integrated backscatter IVUS (IB-IVUS) analysis is currently available only in Japan. Ex vivo validation of this approach has shown that IB-IVUS has high accuracy in detecting plaque composition (19). However, the study of Kawasaki et al. which included 42 coronary arteries from 17 cadavers studied both by IB-IVUS and OCT, showed that both approaches had high sensitivity and specificity in detecting tissue types, but OCT was superior to IB-IVUS (20). Moreover, a recent report including coronary arteries from 14 human autopsy hearts assessed both by OCT and IB-IVUS (360 sections) demonstrated that IB-IVUS had a weak positive predictive value of only 50% but a high negative predictive value of 98.2% in detecting thin cap fibroatheromas (TCFA). In this study, the positive and negative predictive values of OCT were 60.9% and 98.8% respectively. Combining IB-IVUS and OCT, the positive and negative predictive values increased to 100% and 97.7% (21). These findings highlight the additive value of combined imaging in assessing plaque phenotypes. The IB-IVUS have been incorporated in the combined IVUS-OCT system designed by Terumo that is expected to be introduced in the clinical practice in 2025 (22).

### iMap

iMap has been validated in a single histology study showing that this approach is valuable in detecting tissue types (23). However, an *in vivo* study comparing iMAP-IVUS and IB-IVUS showed a weak agreement between the two approaches to characterise plaque composition (24). Today iMap is not clinically available.

## Optical coherence tomography

OCT was introduced for the study of the coronary arteries by professor Fujimoto at the end of the last century (25). This modality has a higher resolution than IVUS allowing more detailed visualization of plaque morphology and lumen pathology than IVUS. The first extensive histology validation of OCT was reported in 2002 and included arterial segments (*n* = 357, 162 aortas, 105 carotids, and 90 coronary arteries) from 90 cadavers. The authors found that OCT enables accurate characterization of all plaque types with high sensitivity (≥95%for calcific plaque, ≥71% for fibrous, and ≥90% for lipid-rich plaques) and specificity (97% for calcific plaque, ≥97% for fibrous, and ≥90% for lipid-rich plaques respectively) (26). Three years later Jang et al. demonstrated the feasibility of OCT *in vivo* and underscored its potential to visualize different plaque phenotypes in patients with an acute and chronic coronary syndrome (27).

**Figure 3:**
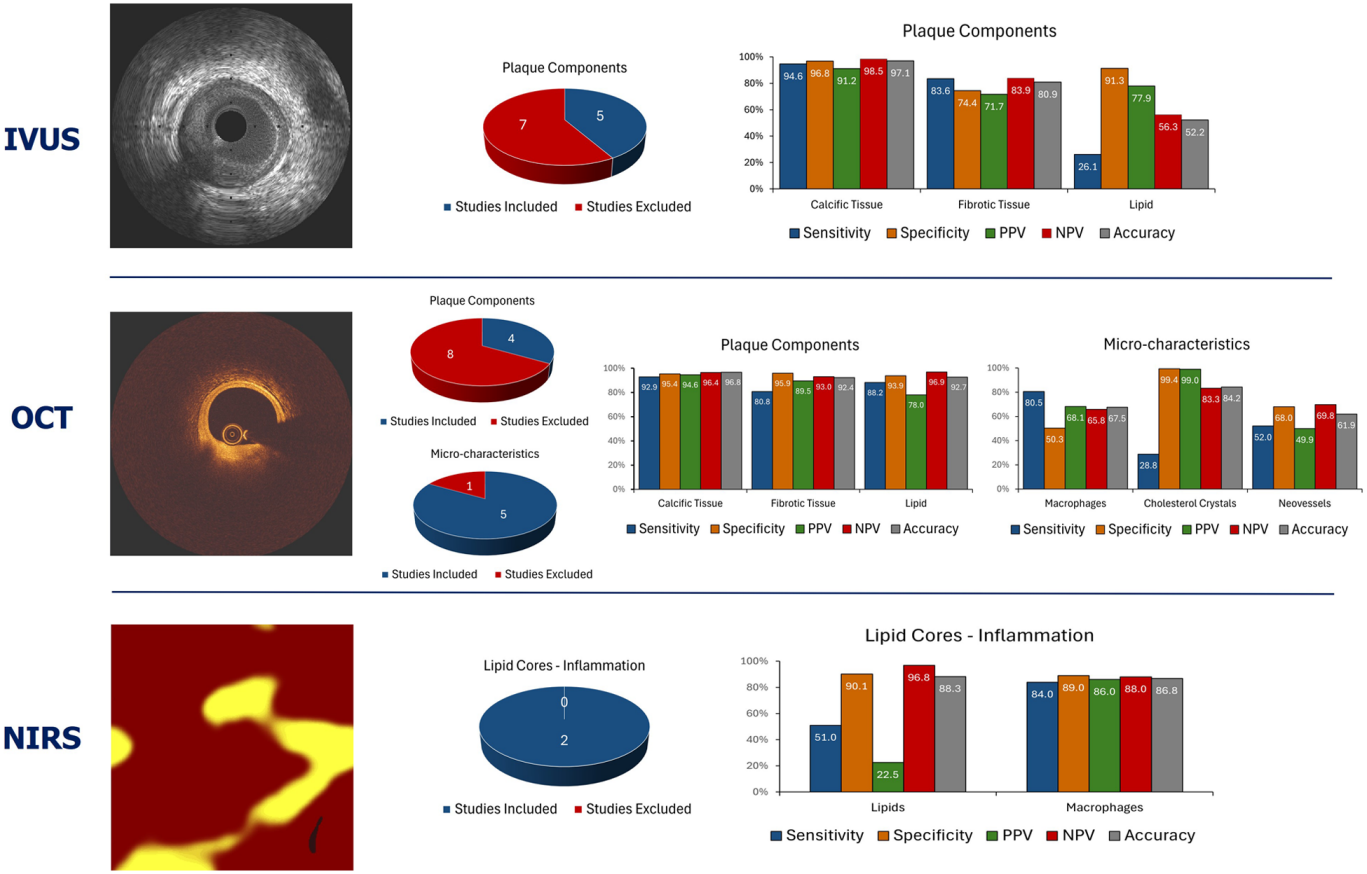
Summary of the average performance of the imaging techniques that are currently available in clinical practice to assess plaque composition. The pie charts summarise the number of studies that tested the performance of each imaging modality in assessing plaque types. The blue colour indicates the studies that allowed measurement of the performance metrics of each modality while the red the studies that did not allow computation of the sensitivity, specificity, positive, negative predictive value, and accuracy for each modality.

Since then several studies have compared and underscored the superiority of OCT over IVUS using histology as reference standard especially in the detection of the lipid compoent (Figure 3) (20, 21, 28–30).

In addition, Kume et al. demonstrated that OCT, in contrast to IVUS, can visualize vessel wall architecture and portray the intima, media, and adventitia layers in disease-free vessels but on the other hand, it is widely acknowledged that OCT can often fail to assess the entire plaque and measure the plaque burden in heavily diseased segments as it has limited signal penetration (1–2 mm within the plaque) (31)

Conversely, the high resolution of OCT has enabled for the first time *in vivo* assessment of plaque microfeatures that have been associated with increased vulnerability and could not be detected by IVUS. Numerous histology studies have shown that the vulnerable plaques that cause events have an increased plaque burden, a large necrotic core component that is covered by a thin fibrous cap, and is rich in macrophages, neovessels, and cholesterol crystals (32–35).

Kume et al. were the first to investigate the performance of OCT in measuring fibrous thickness. The authors included 102 arterial segments from 38 human cadaveric hearts and compared the estimations of OCT against histology. They found a high correlation (*r* = 0.90, *p* < 0.001) but also a significant bias and wide limits of agreement between OCT estimations and histology (24 ± 44 µm). In addition, the authors reported a relatively high inter (13 ± 41 μm) and intra-observer (20 ± 59 μm) variability which can affect the differentiation of thin cap from thick cap fibroatheromas (36). More recent studies have looked into the limitations of OCT to accurately measure cap thickness and showed that the poor definition of the proximal edge of the necrotic core, the presence of macrophages and calcific tissue, as well as deeply embedded lipid tissue can affect the accurate delineation of the fibrous cap thickness (37). This limitation was partially overcome by the design of commercially available software that can automatically detect the fibrous cap border and reproducibly report its thickness (38).

OCT is the only clinically available intravascular imaging modality that can provide information about vascular inflammation. This was demonstrated in the early days of OCT by Tearney et al. who focused on the quantification of the macrophage content in the fibrous cap covering fibroatheromas. In this pivotal study including matched histological and OCT cross-sections the authors showed that increased OCT signal intensity was strongly associated with macrophage density (*r* = 0.83, *p* < 0001) (39). Di Vito et al. few years later, provided further evidence about the value of OCT to identify macrophages and introduced a two-step algorithm for automated macrophages detection. The algorithm included a size filter of structures and an OCT signal intensity metric and was validated against histology in 43 cross sections; the authors found an excellent performance of this approach to identify macrophages (sensitivity and specificity of 100% and 96.8% respectively) (40). However these findings were not confirmed in a larger study including 1,599 matched histological and OCT images. The authors implemented an automated method to detect bright spots in the entire plaque and showed that only 57% of the bright spots corresponded to macrophage accumulation in histology (other causes of bright spots in OCT included fibrous tissue, the interfaces between calcium and fibrous tissue, calcium and lipid deposits and fibrous caps and lipid tissue). Another interesting finding of this analysis was the fact that the macrophages in histology often appear as dark spots in OCT. Nevertheless, when analysis focused on the fibrous caps, they demonstrated a high accuracy of the modality with 94.3% of the bright spots detected by the algorithm corresponding to the presence of macrophages (41). Irrespective of these results the presence of macrophages in OCT seems to provide useful prognostic information as shown in the CLIMA study where inflamed plaques were associated with a higher rate of death or myocardial infarction (42).

Cholesterol crystals appear to play an important role in the biology of vulnerable plaques and studies have shown that they can contribute to their destabilization (35). In OCT, cholesterol crystals appear as thin, linear regions of high intensity (Figure 1). Two studies have examined the efficacy of OCT in detecting cholesterol crystals; the first included 45 matched histological and OCT cross-sections and showed that OCT was capable of correctly identifying these micro-structures with a moderate sensitivity but high specificity (68% and 92% respectively) (43). The second, was a much larger study including 559 matched OCT and histology sections; in this report a cholesterol crystal was defined as linear discrete high-intensity signal within the plaque with sharp borders that was adjacent to low/intermediate intensity tissue. Using this definition, the sensitivity of OCT was low, but the specificity was excellent (25.6% and 100% respectively) for detecting cholesterol crystals. The presence of multiple crystals and the morphological features of adjacent tissues and in particular an overlying fibrous cap were associated with a better accuracy of OCT to identify these plaques (44).

It has been speculated that OCT with its high resolution will be able to identify neo-vessels however, there is limited evidence about its efficacy in identifying these features. In a small histology study of 55 coronary plaques time-domain, OCT was able to detect neovascularization with a moderate sensitivity and specificity of 52% and 68%, respectively (45). There is no data today about the efficacy of frequency domain OCT which has a higher penetration than time domain OCT and is likely to have a better performance in assessing plaque pathology.

Most recently, Gruslova et al. examined the accuracy of seven OCT Core Labs in identifying plaque phenotypes and components. The authors demonstrated an overall moderate agreement between the core labs in assessing plaque features (mean kappa:0.67 ± 0.07); the core labs performed well in identifying fibrotic and calcific plaques, but they had a moderate performance in detecting thick cap fibroatheromas. A weak overall performance was noted between core lab estimations and histology for plaque features associated with increased vulnerability such as lipid tissue, necrotic core, TCFA, macrophages, and calcific nodules (46).

Summarizing the above, it is apparent that OCT is superior to IVUS in assessing plaque composition and biology, but it has inherent limitations in identifying all the plaque features related to increased vulnerability (Figures 2–3; Table 1). To overcomes these limitations polarization-sensitive OCT (PS-OCT) which allows assessment of the polarization state of the reflected OCT signal was proposed to better characterize atherosclerotic plaque components (47). The birefringence and depolarization measurements obtained by PS-OCT seem to provide incremental information about tissue types; however, the added value of this technique over conventional OCT in detecting vulnerable plaques has not been tested yet.

## Near infrared spectroscopy

Near-infrared spectroscopy (NIRS) has extensively been used in chemistry to identify organic substances. It relies on the fact that different molecules can absorb and scatter the NIR light at different intensities and wavelengths. In the study of atherosclerosis, NIRS was used for the first time by Cassis and Lodder to assess plaque composition in rabbits (48), while the first application of this modality in humans was 3 years later by Dempsey et al. who tested its feasibility in detecting lipid tissue in carotids (49).

Moreno et al. provided additional evidence about the efficacy of NIRS to detect the presence of lipid tissue and vascular inflammation in human aortas whereas the first appropriately powered study that examined the value of NIRS to assess plaque composition in the coronary arteries was conducted by Gardner et al. and included coronary segments from 84 autopsy hearts (50, 51). The authors used the first 33 to refine the performance of NIRS to detect necrotic core plaques while the remaining 51 were used to validate it. They demonstrated that the block chemogram that summarizes the probability of the presence of lipid tissue in 2 mm segments was able to identify large necrotic cores—defined as those with circumferential extent >60° located in the superficial plaque with cap thickness <450 μm—with an area under the curve of 0.80 (95% CI: 0.76–0.85) (51, 52).

A subsequent analysis of thise data focusing on the performance of NIRS to characterise plaque phenotype demonstrated a low sensitivity but high specificity of NIRS to identify the presence of fibroatheromas that was attributed to the limited efficacy of the modality in detecting small necrotic cores, while the false positive NIRS estimations were attributed to the lipid component seen in lesions classified as pathological intimal thickening (53). Similar findings were seen in the study of Puri et al. which showed that NIRS was able to detect fibroatheromas with a moderate accuracy (c-index: 0.71) that increased to 0.80 when NIRS was combined with the information provided by IVUS (54). Finally, Inaba et al. explored the efficacy of NIRS in detecting TCFA and showed that a maximum lipid core burden index in a 4 mm segment ≥323—which indicates the fraction of pixels over 1,000 that corresponds to lipid tissue in a 4 mm segment with the largest lipid component—had an excellent accuracy in detecting this phenotype (AUC: 0.84) (54).

Summarizing the findings of these studies, it is apparent that NIRS has a high diagnostic accuracy in identifying lipid tissue; however, this modality cannot visualize the lumen and vessel wall, quantify the plaque burden, and provide depth information about the location of the lipid tissue in the plaque (Figure 3; Table 2). To overcome these limitations NIRS has been combined with an IVUS probe (TC Imaging System™ and Makoto Intravascular Imaging System™, Infraredx) in a hybrid NIRS-IVUS system; this prototype can assess lumen morphology and atheroma burden and in a recent histology study it has been shown that the circumferential location of the lipid tissue given by NIRS, and the pixel intensity of the plaque provided on IVUS allows accurate estimation of the lipid tissue distribution in the plaque (55).

**Table 2:** Efficacy of invasive imaging modalities that provide an assessment of the biochemical composition of the plaque and of plaque biology in characterizing plaques characteristics using human histology data as reference standard.

Study	Objectives	Sample size	Results
NIRS			
Moreno et al. (50)	To assess the efficacy of NIRS in detecting vulnerable plaque features and in particular lipid pools, atheromas with a fibrous cap thickness <65 µm and inflammatory cells	199 samples from 5 human aortas were assessed by NIRS and matched with histology	• NIRS was able to detect lipid pools with a high sensitivity, specificity, positive and negative predictive value (90%, 93%, 90% and 93% respectively) • The modality had also a high diagnostic accuracy in detecting thin-cap fibroatheromas (sensitivity 77%, specificity 93%, positive predictive value 68% and negative predictive value 95%) • The sensitivity of NIRS to detect inflammatory cells was 84%, the specificity 89%, the positive predictive value 86% and the negative predictive value 88%
Gardner et al. (51)	To assess the efficacy of NIRS in detecting lipid-rich plaques in coronary arteries of human cadavers	212 coronary segments from 84 autopsied human hearts were assessed by NIRS; 33 hearts were used to refine the performance of NIRS and the remaining to evaluate its accuracy	• In the studied segments blocks of 2 mm were generated; a histology cross sections taken from each block was compared with the estimations of the block chemogram indicated the presence of lipid in NIRS • Yellow block chemograms accurately predicted the presence of lipid cores with an AUC of 0.80 • The LCBI which indicates the per mille of the lipid core in the studied vessels was able to identify arteries containing lipid-core plaques with an AUC of 0.86
Kang et al. (53)	To assess the ability of grayscale IVUS and NIRS to detect FAs	1,943 histological cross-sections from 103 human coronary arteries from 56 autopsied hearts were matched with NIRS and IVUS imaging data	• The presence of superficial attenuated plaques in IVUS had a sensitivity, specificity, positive, negative predictive value for detecting FAs of 36%, 96%, 66%, 87% respectively • The sensitivity, specificity, positive and negative predictive value of the block chemogram in NIRS to detect FAs were 47%, 94%, 65% and 89% respectively • Combined IVUS and NIRS imaging improved the positive predictive value of intravascular imaging to detect FA compared to standalone IVUS or NIRS (84% vs. 66% vs. 65%; *p* < 0.001)
Puri et al. (54)	To test the performance of IVUS-derived plaque features and of NIRS in assessing plaque phenotype	106 lesions detected in 116 human coronary arteries of 51 autopsied hearts were assessed by NIRS and IVUS included in the study	• The plaque burden (OR: 2.26, 95%CI: 1.11–4.58) and remodelling index (OR: 2.71, 95%CI: 1.23–6.02) on IVUS and the NIRS-derived LCBI (OR: 2.15, 95%CI: 1.11–4.15) were independent predictors of FA • The c-index of IVUS, NIRS and combined NIRS-IVUS in detecting FAs was 0.760, 0.712 and 0.800
Inaba et al. (98)	To investigate the efficacy of IVUS and NIRS in characterizing TCFA lesions	271 atherosclerotic lesions from 107 human coronary arteries derived from 54 autopsy hearts were imaged with NIRS-IVUS and matched with histology	• NIRS-derived maxLCBI_4mm_ ≥ 323, plaque burden ≥ 69% and remodelling index ≥1.07 were able to detect TCFA with an AUC of 0.84 (80% sensitivity and 85% specificity), 0.87 (90% sensitivity and 75% specificity) and 0.84 (80% sensitivity and 79% specificity) respectively • Combined IVUS-NIRS imaging had better a accuracy in predicting TCFA than standalone IVUS or NIRS (91% vs. 69%)
NIRF imaging			
Albaghdadi et al. (62)	To characterize plaque components associated with positive NIRAF signal	15 human carotid endarterectomy specimens were imaged using fluorescence reflectance imaging and compared with histology	• NIRAF-positive areas corresponded to lipid areas (*r* = 0.53, *P* = 0.023) and intraplaque haemorrhages (*r* = 0.48, *P* = 0.043) to a similar extent • NIRAF correlated with the presence of macrophages (*r* = 0.92, *P* = 0.001) and there was an inverse association with ACTA2 + smooth muscle cells (*r* = −0.72, *P* = 0.002)
Kunio et al. (63)	To identify coronary plaque features associated with autofluoresence	Ex-vivo OCT-NIRAF was used to assess the coronary arteries of 23 fresh human cadavers	• 58% of the NIRAF-positive areas were rich in ceroids and had an intraplaque haemorrhage in histology, 6.3% of the NIRAF-positive areas had only intraplaque haemorrhage and 33.6% had only ceroids • The presence of ceroids was strongly associated with NIRAF-positive regions than the intraplaque haemorrhages (Dice similarity coefficient: 0.072 ± 0.096 vs. 0.060 ± 0.090, *P* < 0.01).
FLIm imaging			
Fatakdawala et al. (66)	To evaluate the efficacy of combined FLIm and IVUS in detecting plaque morphology	Co-registered FLIm and IVUS data (*n* = 87 cross-sections) from 16 coronary arteries were correlated with histology	• The sensitivity, specificity and positive predictive value of the combined FLIm-IVUS imaging (89%, 99%, 89%) were significantly higher than standalone IVUS (45%, 94%, 61%) or FLIm (70%, 98%, 88%).

AUC, area under curve; FA, fibroatheroma; FLIm, fluorescence lifetime imaging microscopy; IB, integrated backscatter; IVUS, intravascular ultrasound; LCBI, lipid core burden index; LDL, Low density lipoprotein; maxLCBI4 mm, Maximum LCBI in 4 mm segment; NIRS, near infra-red spectroscopy; NIRAF, near-infrared autofluorescence; OCT, optical coherence tomography; TCFA, thin capsule fibroatheroma.

More recently a hybrid OCT-NIRS probe was introduced that has undergone a first in-man study (56). Both systems have FDA approval for the detection of patients at risk of suffering MACE.

## Near-infrared fluorescence imaging

Near-infrared fluorescence imaging (NIRF) has emerged as a translational intravascular modality for assessing plaque pathobiology. Imaging relies on the injection of specialized imaging agents which can bind molecules associated with specific biological processes and can fluoresce after being irradiated with NIR light.

Several animal studies have provided unique insights into the efficacy of this modality in assessing plaque activity. Today it is known that NIRF can detect, depending on the injected marker, macrophages accumulation, ICAM-1 an unpolymerized type I collagen, protease activity, and fibrin following stent implantation (57–59). Moreover, the study of Aikawa et al. underscored the value of NIRF to detect osteogenesis in mice using the activatable marker OsteoSense750 and reported a link between vascular inflammation and osteogenic activity in human plaques obtained following carotid endarterectomy (60). In addition, the BRIGHT-CEA study involving human specimens from carotid endarterectomy has shown that NIRF imaging, after injection of indocyanine green can detect an impaired endothelial integrity, including disrupted fibrous caps, areas of neovascularization, macrophages, lipid tissue, and intraplaque haemorrhages (57).

The first-in-man study examining the feasibility of NIRF imaging used a hybrid OCT-NIRF catheter and demonstrated that some plaques have the ability to auto fluoresce (NIRAF) without the need to inject activatable markers (61). Recent histology studies have provided further insights into the phenomenon with consistent results. The study of Albaghdadi et al., which included 15 human carotid endarterectomy specimens, demonstrated that NIRAF was associated with intraplaque haemorrhage (*r* = 0.48, *P* = 0.043) and lipid—specifically insoluble lipid or ceroid (*r* = 0.53, *P* = 0.023) (62). Similar were the findings of another study from the same research group that included coronary arteries from 31 fresh human donated hearts which showed that autofluorescence was associated with the presence of intraplaque haemorrhage and insoluble lipid or ceroid and that the association was stronger between autofluorescence and ceroid than intraplaque haemorrhage (63). The NIRF studies highlighted are summarised in Table 2.

NIRF has been combined with IVUS or OCT catheters in hybrid intravascular systems that are expected to enable anatomical and biological characterization of coronary atheroma, Canon Inc., Tokyo, Japan has invested in the commercialization of NIRF-OCT imaging which is anticipated to become clinically available in the following years.

## Fluorescence lifetime imaging

Fluorescence lifetime imaging (FLIm) has been introduced to assess the biochemical composition of the superficial plaque. Imaging with this modality relies on the fact that different molecules can fluoresce for a specific time period after being irradiated with NIR light. Animal studies confirmed that measurement of the fluorescence time allows assessment of plaque composition and vascular inflammation (64, 65).

The potential of FLIm imaging is also supported by histology studies in human cadaveric hearts. In the study by Fatakdawala et al., combined IVUS and FLIm were used to characterise plaque morphology in 87 histological sections from 16 human coronary arteries; the authors showed that FLIm imaging had a superior sensitivity, specificity, and accuracy in assessing plaque phenotypes than standalone IVUS (70%, 98, 88% vs. 45%, 94% and 62% respectively). Combined FLIm-IVUS imaging outperformed standalone imaging in assessing plaque morphology (sensitivity 89%, specificity 99%, and accyracy 89%, respectively) (Table 2) (66). These results were also confirmed by a histology study including 47 segments obtained from human hearts that were imaged by a multimodal FLIm-OCT system. FLIm-OCT imaging was able to accurately detect 89.4% of the plaques underscoring the potential of hybrid-FLIm based imaging to assess plaque phenotypes (67).

In line with these findings a recent report including 32 human coronary artery segments assessed by combined IVUS-FLIm showed that FLIm was capable of accurately detecting superficial calcium (ROC-AUC: 0.90) and macrophages with high accuracy (ROC-AUC: 0.94) (68). The efficacy of FLIm to detect vascular inflammation was also confirmed by the study of Rico-Jimenez et al. that included 80 fresh post-mortem coronary segments from 23 autopsy hearts and showed that FLIm imaging was very accurate in identifying macrophages in the superficial plaque with an accuracy of 91.5% (69). The relevant studies of FLIm are summarised in Table 2.

FLIm imaging has been combined with OCT in a hybrid multimodality imaging probe developed by the Dotter Inc Seoul Korea that has been recently tested in clinical practice (NCT04835467). There is also a combined IVUS-FLIm catheter system that has an external diameter of 3.7Fr and is pulled back at a maximum speed of 4 mm/s. Imaging with this system requires blood clearance with bolus flushing; therefore, the maximum length that can be studied with this system is rather limiting. This constraint as well as the large diameter of the catheter has not enabled its application in the clinical arena (70).

## Intravascular photoacoustic imaging

Intravascular photoacoustic (IVPA) imaging relies on the processing of the acoustic signal that is produced after the thermal expansion of molecules that have been irradiated with laser-light. In contrast to the NIRS, NIRF, and FLIm, IVPA provides depth information of tissue distribution by measuring the time interval between tissue irradiation and the IVPA signal.

Several experimental and animal studies have demonstrated the potential of IVPA to detect lipid tissue, collagen and metallic struts, as well as vascular inflammation and endothelial integrity when imaging is performed after injection of specific agents that are able to bind molecules that regulate plaque biology (71–75).

There is limited evidence about the performance of IVPA to characterize plaque morphology using ex vivo human data as reference standard. Reports involving a single coronary artery have shown the feasibility of this modality to detect lipid tissue (76), while Arabul et al. has shown that IVPA can identify intraplaque haemorrhages in atherosclerotic carotid plaques obtained following endarterectomy (77).

IVPA has been combined with IVUS imaging in hybrid IVPA-IVUS systems that enables evaluation of lumen and plaque dimensions and characterization of its composition. Several prototypes have been presented over the last few years for combined IVPA-IVUS imaging, however none of them has reached the clinical practice (78, 79). Kaminari Medical B.V., Rotterdam Netherlands is a recently developed company that aims to overcome limitations of previous revisions and design an hybrid IVPA-IVUS catheter for clinical applications. (https://kaminarimedical.com).

## Discussion

From the early days of intravascular imaging, histology-based studies have been used to test the performance of existing and emerging modalities in evaluating vessel wall pathology and appreciate their potential in the clinical practice and research. These studies by identifying the advantages and limitations of each modality have helped in the evolution of intravascular imaging and guide the development of novel approaches that will outperform the existing techniques.

Today it is acknowledged that none of the available modalities is able to provide a complete and detailed visualization of plaque pathology. Therefore, there is a trend these days to design hybrid intravascular imaging systems that will incorporate two different imaging probes with complementary strengths for more detailed characterization of plaque pathobiology. The combined IVUS-OCT system as well as the NIRS-IVUS, OCT-NIRS, OCT-NIRF, FLIm-OCT and IVPA-IVUS are typical examples (34, 80, 81).

**Figure 4:**
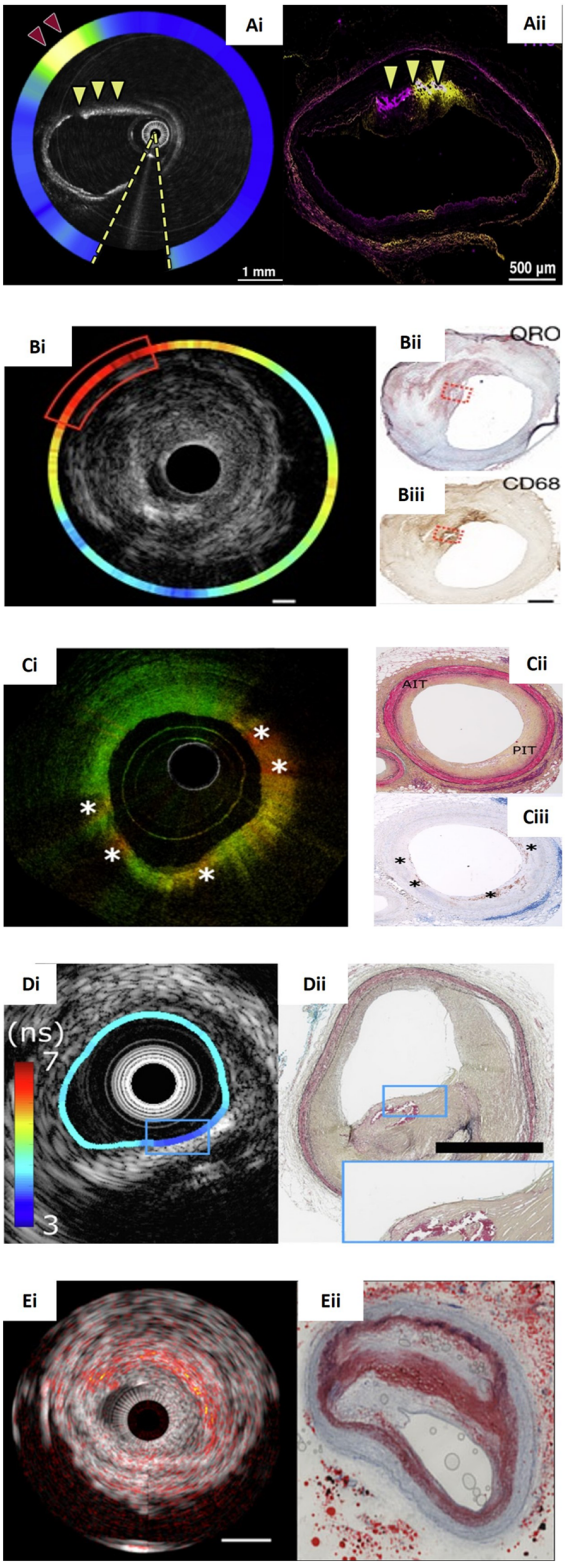
Panorama of hybrid intravascular imaging modalities and the corresponding histology. **(Ai)** An OCT-NIRF frame obtained after indocyanine green (ICG) injection showing increased uptake at (red arrowhead) in a lesion with intimal and medial calcification (yellow arrowheads) that was also confirmed by the corresponding fluorescence microscopy image **(Aii)**. **(Bi)** NIRF-IVUS image showcasing high ICG-concentrations (red box) spatially related to a lipid and infiltrated macrophages as seen in the corresponding Oil Red O (ORO)-stained **(Bii**; red dotted box**)** and CD68-stained histological section **(Biii**; red dotted box**)** respectively. **(Ci)** An OCT-FLIM imaging with increased 540-nm lifetime values that correspond to pathological intimal thickening as seen in Movat's pentachrome-stained histology **(Cii**; PIT**)** and macrophages accumulation (*) that were confirmed by CD68-stained histology **(Ciii)**. **(Di)** FLIM-IVUS cross-section demonstrating the presence of lipid tissue detected by FLIm in a calcified plaque confirmed by IVUS and the corresponding histology section stained with Movat's pentachrome showing a mixed plaque **(Dii)**. **(Ei)** IVPA-IVUS frame portaying a lipid-rich plaque indicated with red-orange overlay. The corresponding ORO-stained histology confirms a large necrotic core **(Eii)**. Image **(Bi–Biii)** courtesy of Rauschendorfer et al. (10.1038/s44325-024-00016-8). Image **(Ci–Ciii)** courtesy of Li et al. (10.1117/1.JBO.27.7.076005). Image **(Ai,Aii)**, **(Di,Dii)**, **(Ei,Eii)** courtesy of Tufaro et al. (10.1016/j.jcin.2024.07.007).

Preliminary validations studies of these systems using histology as reference standard have provided proof of the consensus showing that these approaches outperform standalone intravascular imaging systems in assessing plaque types (Figure 4) (55, 82). Histology is the gold standard for assessing plaque composition, however, the obvious requirement for intact coronary vessels to be used in these studies limits validation to model systems only, all of which have their drawbacks. Firstly, different vascular beds have different characteristics; for example, the carotid vessels have different elastic and smooth muscle composition to coronary vessels. In addition, cadaveric analysis from anatomy departments is compromised by their prior fixation and the fresh vessels from explanted recipient hearts or even post- mortem are compromised due to the extended period prior to sampling.

Other limitations of the studies assessing the performance of standalone intravascular imaging modalities include the fact that: (1) they are not appropriate powered, (2) they don't have a specific primary endpoint, (3) they often include a small number of matched frames that do not allow us to draw safe conclusions, (4) the matching of the intravascular imaging and histology data can be challenging and affect the final results, and (5) the tissue shrinkage that occurs during histological preparation that does not allow us to accurately test the performance of intravascular imaging to quantify plaque burden and composition. Moreover, in most of the histology studies a single histological staining was used that does not enable a complete evaluation of plaque pathology and is likely to influence image interpretation. Finally, the reproducibility of the clinicians and the histopathologists who performed the analyses is not always reported even though it can influence the results (83, 84).

It is therefore essential to standardize analyses protocols and perform large prospective histopathological based studies that will be appropriately powered and will facilitate—using landmarks—the intravascular imaging and histology sections matching and will make use of multiplex immunohistochemistry and 3D based histology so as to appreciate the full potential of each modality to assess plaque pathology. This approach will allow us to better understand the potential of the existing catheters, identify their limitations, and design future prototypes that will combine multiple imaging probes to address the current clinical needs (i.e., optimal PCI planning and vulnerable plaque detection) and improve clinical outcomes.

These datasets can be also used to train and test the performance of deep learning (DL) classifiers that will allow fast, accurate and reproducible characterization of plaque phenotypes. Proof of concepts studies have highlighted the potential of DL methods in the analysis of intravascular imaging data (55, 85–87). However, the small number of histological sections included, the use of a single stain and limitations in the co-registration of intravascular imaging and histology have not allowed us to explore the full potential of DL in this setting. There is therefore an unmet need to generate large optimal intravascular-histological imaging data that will be stained with multiple stains enabling thorough assessment of plaque characteristics and use these to develop efficient DL solutions that will allow enhanced tissue characterization reliable assessment of plaque vulnerability and more accurate risk stratification.

This strategy may also be helpful in the optimization of the deep learning solutions that have been introduced to analyze computerized tomography coronary angiography (CTCA) data and unlock the full potential of CTCA in the study of atherosclerosis, enable more precise detection of vulnerable plaque, and quantification of the lumen and vessel dimensions that are essential in treatment planning (88–91).
